# Investigating Sex‐Biased Dispersal in a Vulnerable Marine Invertebrate, the European Spiny Lobster (*Palinurus elephas*)

**DOI:** 10.1002/ece3.73617

**Published:** 2026-06-07

**Authors:** Laura Benestan, Alicia Dalongeville, Raquel Goñi, Sandra Mallol, David Díaz, Oscar Puebla, Stéphanie Manel

**Affiliations:** ^1^ CEFE Univ Montpellier, CNRS, EPHE‐PSL University, IRD Montpellier France; ^2^ MARBEC Univ Montpellier, CNRS, IFREMER, IRD Montpellier France; ^3^ Instituto Español de Oceanografía (IEO, CSIC) Centro Oceanográfico de Baleares Palma de Mallorca Spain; ^4^ Leibniz Centre for Tropical Marine Research (ZMT) Fahrenheitstraße 6 Bremen Germany; ^5^ Institute for Chemistry and Biology of the Marine Environment, Carl von Ossietzky Universität Oldenburg Oldenburg Germany; ^6^ Institut Universitaire de France Paris France

**Keywords:** European spiny lobster, fisheries management, sex‐biased dispersal, sex determination system

## Abstract

Does dispersal differ between the sexes? This question, anchored in a body of rich theoretical literature, has received little empirical attention in marine invertebrates. Yet, dispersal is a key ecological process with profound implications for species management and conservation. In this study, we investigated sex‐biased dispersal in the European spiny lobster ( *Palinurus elephas* ) by sampling females and males from six marine reserves and their surroundings in the northwestern Mediterranean. By genotyping 180 individuals at 8390 markers, we found that both sexes exhibited panmixia. Genetic differentiation estimates did not differ significantly between sexes, despite a tendency toward slightly higher and more geographically structured estimates in females. Additionally, we identified 72 sex‐linked markers with significant differences in heterozygosity between females (fully heterozygote) and males (fully homozygote). These markers not only allowed the sex assignment of 61 individuals of unknown sex but also provided the first evidence for a ZZ/ZW sex determination system in *P. elephas* . Beyond their fundamental insights, these sex‐linked markers hold strong applied potential for species management, particularly in fisheries where sex‐specific regulations exist. Our findings underscore the power of genomic markers to study sex‐biased dispersal, elucidate sex determination systems, and facilitate sex assignment, with important implications for species conservation and management.

## Graphical abstract

Does dispersal differ between the sexes? Our findings underscore the power of genomic markers to study sex‐biased dispersal, elucidate sex determination systems, and facilitate sex assignment, with important implications for species conservation and management.

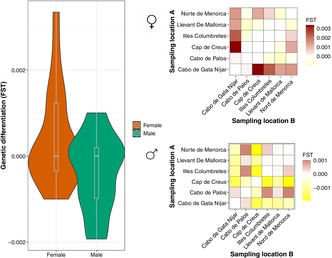

## 1 Introduction

Dispersal, that is, any movement of individuals or propagules that has potential consequences for gene flow across space (Clobert et al. 2012), is a central and multifaceted biological process that has important implications at multiple levels: for survival, growth and reproduction; for population structure and dynamics, and for population resilience and adaptation (Baguette et al. 2012; Cayuela et al. 2018). Because the costs and benefits of dispersal can differ between the sexes, sex‐biased dispersal—where one sex is more prone to disperse than the other—is common in both terrestrial and marine organisms (reviewed in Li and Kokko 2019; Prugnolle and de Meeus 2002). One prominent eco‐evolutionary hypotheses put forward to explain sex‐biased dispersal is intrasexual selection, which posits that the sex that experiences greater competition is more likely to disperse (Dobson 1982). According to this hypothesis, female mate choice and intense male–male competition for females in polygamous mating systems are expected to promote male dispersal (Greenwood 1980). Polygamous mating systems, including both polygyny (i.e., males mating with multiple females) and polyandry (i.e., females mating with multiple males) have been documented in genetic studies reporting multiple paternity (reviewed in Palaoro and Beermann 2020). Another, nonexclusive hypothesis to explain sex‐biased dispersal is inbreeding avoidance, which predicts that the risk of mating with relatives decreases when one sex disperses more than the other (Pusey 1987). However, empirical analyses suggest that the influence of inbreeding avoidance on sex‐biased dispersal is relatively weak (Pike et al. 2021).

In the marine realm, research on sex‐biased dispersal has predominantly focused on vertebrates, including fish (Hutchings and Gerber 2002), sharks (Day et al. 2019; Pardini et al. 2001; Phillips et al. 2021; Portnoy et al. 2015), sea turtles (Clusa et al. 2018), and marine mammals (Ball et al. 2017; Möller and Beheregaray 2004; Nykänen et al. 2018). In contrast, invertebrates have received comparatively little attention (Teske et al. 2012; Durand et al. 2019; King et al. 2005). A notable exception is the study by Teske et al. (2012), which revealed a sex‐specific genetic structure in the brown mussel ( *Perna perna* ) based on mitochondrial DNA markers. However, this pattern was not attributed to sex‐biased dispersal but rather to differential selective pressure between the sexes, as this species disperses passively, and no sex‐specific structure was detected at another nuclear marker. Sex‐biased dispersal occurs when one sex tends to move away from its birthplace more than the other, influencing patterns of gene flow and population structure. In contrast, sex‐specific selective pressures arise when males and females experience different evolutionary forces affecting survival or reproduction, often leading to sexual dimorphism or differences in life‐history traits. While the two processes can interact, they are conceptually distinct: dispersal concerns where individuals go, whereas selective pressures concern which individuals survive or reproduce more successfully (Li and Kokko 2019). More recently, Peres et al. (2021) investigated sex‐biased dispersal in the tube‐building amphipod ( *Cymadusa filosa* ) and found no differences in genetic structure between females and males. Both studies focused on passive dispersers, but other invertebrates, such as crabs or lobsters, have the capacity for active dispersal in the adult stage. As with terrestrial arthropods such as insects, these species may experience sex‐biased dispersal, highlighting the need for further research on this topic in marine invertebrates, particularly because many marine invertebrate fisheries are managed using sex‐specific regulations (Baines et al. 2017; Dudaniec et al. 2022; Durand et al. 2019; Johnstone et al. 2012).

Sex‐biased dispersal can be detected by quantifying differences between females and males in relatedness and genetic differentiation using bi‐parentally inherited markers such as single nucleotide polymorphism (SNPs) or microsatellites. Robust genetic data rely heavily on large sample sizes and the use of a sufficient number of polymorphic markers capable of resolving genetic differentiation. This approach allows for the identification of sex‐biased dispersal within a single generation if genetic sampling is conducted after dispersal (and ideally before breeding) in both sexes (Goudet et al. 2002; Prugnolle and de Meeus 2002). If sex‐biased dispersal does not persist across generations, its genetic signature is expected to fade as offspring inherit one allele from each parent at these markers. In theory, sex‐biased dispersal can also be inferred over long timescales by comparing the genetic structure at uniparental inherited markers (e.g., mtDNA, Y‐linked markers) with that at biparental inherited markers. Since only one sex transmits the genome at uniparentally inherited markers, whereas both sexes contribute to biparentally inherited markers, differences in genetic structure between these marker types could indicate sex‐biased dispersal. However, this approach has notable limitations. Differences in dispersal between the sexes can be confounded by differences in mutation rates and effective population size (*N*_e_) between marker types (Chesser and Baker 1996), or by the influence of mating systems (Shaw et al. 2018), complicating the interpretation of the results.

Dispersal and the resulting gene flow act as homogenizing forces that counteract genetic drift across the genome, even with only a few numbers of migrants per generation in an infinite island model of population structure (Saastamoinen et al. 2018; Wright 1931). Population genetic theory predicts that the less dispersing sex should have higher levels of relatedness among individuals, lower genetic diversity, and stronger population genetic structure (Goudet et al. 2002). The less dispersing sex is also expected to have a lower contemporary effective population size (*N*_e_), as genetic structure tends to reduce *N*_e_ (Whitlock and Barton 1997). Because effective population size influences population viability, it plays a critical role in wildlife management and conservation planning (Allendorf et al. 2010). Accurate estimates of *N*_e_ are essential for the management of exploited species vulnerable to overfishing, such as the European spiny lobster ( *Palinurus elephas* ).

The European spiny lobster is an iconic species of the Northeast Atlantic and Mediterranean. Adult lobsters can migrate up to 70 km seasonally (Cau et al. 2019; Follesa et al. 2009; Goñi and Latrouite 2005; Moland et al. 2025) while pelagic larvae can be dispersed by ocean currents for 4–6 months before settlement (Follesa et al. 2009; Muñoz et al. 2021). In the Mediterranean Sea, *Palinurus elephas* exhibits a seasonal complex reproductive cycle, with mating and egg laying primarily occurring between July and October and a peak of berried females observed in late summer and early autumn. Reproduction is once a year, with females carrying one to three clutches of eggs under the abdomen that incubate for around 5 months before hatching in late winter to spring. The planktonic larvae, called phyllosoma, spend several months in the water column before settling as puerulus in early summer, typically between June and September (Goñi and Latrouite 2005). Behavioral differences between the sexes are evident in catch‐per‐trap data: males tend to be solitary or found with a single female and exhibit agonistic interactions with conspecifics, whereas females tend to cohabit in traps with other lobsters, regardless of sex (Goñi et al. 2003). The conclusion obtained in trap experiments reflects the natural behavior during mating season. Males have agonistic interactions with other males and tend to be in the company of at least one female (often two or three females), while females are more likely to cohabit with other congeners. Sexual selection also drives differences in body size, with males being larger than females (Goñi and Latrouite 2005) and exhibiting a faster growth rate (Bevacqua et al. 2010). Microsatellite analyses have shown that *P. elephas* forms a panmictic unit throughout the Mediterranean basin (Babbucci et al. 2010; Palero et al. 2011). This finding was further confirmed by a recent population genomic study reporting complete panmixia in the northwestern Mediterranean (Benestan et al. 2021). However, sex‐specific population structure has not been investigated in this species.

In this study, we aim to first test whether dispersal in *P. elephas* differs between the sexes. If *P. elephas* follows a polygamous mating system, intrasexual competition for local resources and mating opportunities would predict sex‐biased dispersal. We then leverage our dataset to elucidate the sex determination system of *P. elephas* and explore the potential of sex‐linked markers for sex assignment.

## 2 Methods

### 2.1 Sampling and Genotyping

**FIGURE 1:**
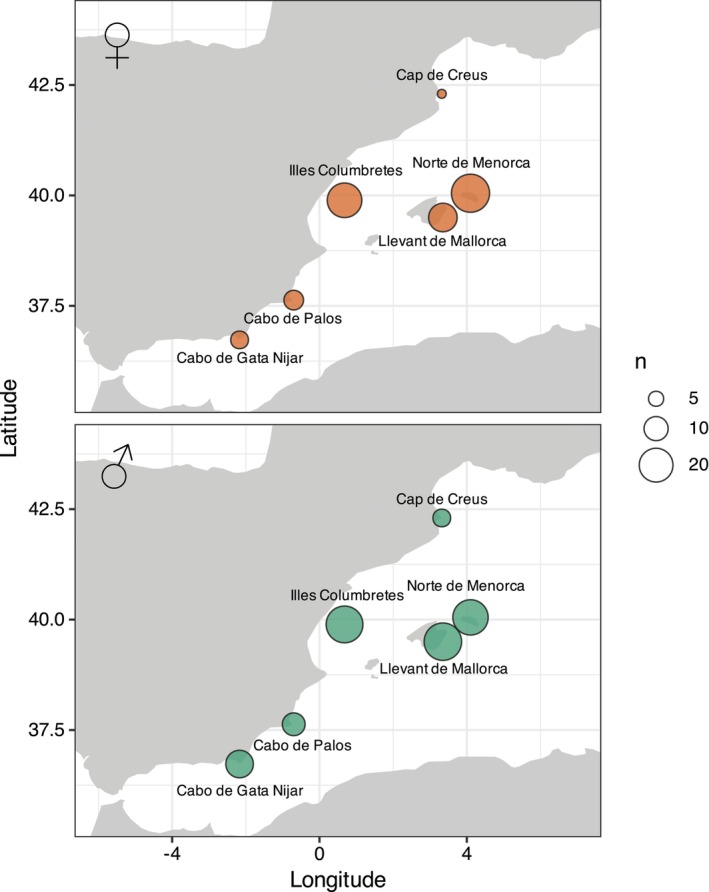
Sampling design. Map showing the distribution of sampling locations of the two sexes and sample size in *Palinurus elephas* .

To investigate sex‐specific dispersal, we subsampled the *P. elephas* dataset from Benestan et al. (2021) to focus only on individuals that were sexed based on morphological attributes: presence of a spermatophore at the base of the last two pairs of walking legs in males and claw‐shaped end of the fifth pair of walking legs in females. In total, 79 females and 101 males (180 individuals) from six marine reserves in the northwestern Mediterranean were finally considered (4–26 samples per reserve; Figure 1, Table 1). Individuals not sampled within a reserve were assigned to the nearest reserves (mean distance to the nearest reserve 36 km, maximum 90 km). The sampling design also included the Balearic Islands, which is the most productive Spanish fishery for *P. elephas* (Quetglas et al. 2004). Sampling was conducted on adult individuals, that is, after larval dispersal and presumably after adult dispersal as well. Samples were collected between March and November during the premating and mating periods, when spiny lobsters migrate from deep waters to shallower areas for reproduction. Thus, if dispersal is sexually biased, we expected this to be reflected in our data through differences in relatedness, genetic structure and effective population size between the sexes. Further details of sample processing can be found in Fietz et al. (2020) and Benestan et al. (2021). Briefly, genomic DNA was extracted from pleopods using the ReliaPrep gDNA Tissue Miniprep System (Promega GmbH, Mannheim, Germany) according to the manufacturer's protocol for animal tissue. DNA concentration was quantified using a Qubit 2.0 fluorometer (Thermo Fisher Scientific Inc., Waltham, USA). Samples were then genotyped using Diversity Array Technology (DArT) sequencing, a variant of function (Kilian et al. 2012), through an outsourcing company.

**TABLE 1:** Number of female and male lobsters sampled in and around six marine reserves, longitude, latitude (decimal degrees) and year of establishment of each reserve.

Marine reserve	# Females	# Males	Longitude	Latitude	Year of establishment
Cabo de Gata Níjar	7	7	−2.07	36.87	1987
Cabo de Palos	7	17	−0.65	37.65	1995
Cap de Creus	4	6	3.23	42.30	1998
Illes Columbretes	21	24	0.67	39.88	1990
Llevant de Mallorca	14	25	3.40	39.79	2007
Nord de Menorca	26	22	4.05	40.07	1999

*Note:* Individuals were assigned to one of the six reserves considered in this study based on their distance to the nearest reserve boundary.

### 2.2 SNP Filtering

We extracted the 25,230 putatively neutral loci and the 833 outlier loci identified using pcadapt (Luu et al. 2017) from the dataset published by Benestan et al. (2021). Pcadapt is an outlier detection method that analyses genotype data at the individual level, making it suitable for our sampling, which is only loosely clustered around six reserves. Putative neutral SNPs were previously filtered based on minimum allele frequency (MAF, > 0.05), sequence coverage (between 10× and 100×), missing data (< 0.2 per locus), and linkage disequilibrium (*R* ^2^ < 0.8, Table S1) in 243 individuals. An exploratory principal component analysis was performed, and two highly related individuals were detected. These two individuals were removed from subsequent analyses, resulting in a final sample of 241 individuals. From this sample, we re‐filtered the putatively neutral dataset for the subset of 180 individuals with known sex information. Using the *dartR* package (Gruber et al. 2018), we retained SNPs with a 95% genotyping rate (23,814 SNPs), a MAF > 0.05 (9931 SNPs), and removed markers indicating departures from Hardy–Weinberg equilibrium as well as monomorphic SNPs. We then used this subset of putatively neutral SNPs (8390 SNPs) to compare relatedness, population structure, and *N*_e_ between the sexes.

### 2.3 Test for Sex‐Biased Dispersal

Population structure was first assessed using discriminant analysis of principal component (DAPC), without incorporating any prior information (e.g., sampling location) to guide clustering. To do this, we ran the *find.cluster* function, available in the *adegenet* package (Jombart et al. 2010) to determine the optimal number of clusters (*K*) in our dataset based on the Bayesian Information Criterion. This *K*‐mean algorithm sequentially increases the number of clusters after transforming the data using a principal component analysis (PCA). To avoid overfitting, we retained 17 axes according to the alpha score output, as recommended by the authors of *adegenet* (Jombart et al. 2010).

We estimated Loiselle's relatedness index using GenoDive (Meirmans and Van Tienderen 2004) to assess the difference in relatedness between the sexes. This index was selected because it corrects for small sample sizes with less bias than other coefficients (Wang 2017). Previous analyses have shown that the Loiselle relatedness index correlates well with other commonly used relatedness coefficients (Benestan et al. 2021). To evaluate genetic differentiation, the Weir and Cockerham (1984) index (*F*_ST_) between marine reserves was calculated separately for each sex using the *hierfstat* package in R (Goudet and Jombart 2015). Bootstrapped confidence intervals were estimated using 100 iterations (argument *iteration.ci*; resampling with replacement of markers). The significance of *F*_ST_ estimates was assessed using Genodive (Meirmans and Van Tienderen 2004). We note that the test for sex‐biased dispersal implemented in *hierfstat* (Goudet and Jombart 2015) is not applicable to our dataset due to the trade‐off between the number of samples and the number of markers considered. We therefore performed an AMOVA including both sampling location and sex as hierarchical factors, focusing on the partitioning of genetic variance between the two sex groups (males vs. females) rather than among sampling locations (Table 2). Finally, we estimated the contemporary effective population size (*N*_e_) separately for females and males under the assumption of random mating, using the linkage disequilibrium method implemented in NeEstimator (Do et al. 2014).

**TABLE 2:** Analysis of Molecular Variance (AMOVA) showing the partitioning of genetic variation among sexes and sampling locations.

Source of variation	df	Sum of squares	%	*p*
Between sexes	5	5879.483	−0.0214	0.8251
Between locations within sexes	6	7132.773	0.0508	0.0599
Between samples within locations	168	196992.073	5.2473	0.0009
Within samples	180	190011.000	94.7232	0.0009
Total	359	400015.328	100.000	—

### 2.4 Identifying Sex‐Linked Markers

The function *gl.sex.linked* from the *dartR* package in R was used to identify sex‐linked markers in the dataset (Robledo‐Ruiz et al. 2023). This function compares allele frequencies or genotype patterns between males and females for each locus to detect sex‐specific patterns. Loci showing alleles that are predominantly or exclusively present in one sex are classified as gametologous (X‐ or Y‐linked in XY systems, Z‐ or W‐linked in ZW systems), whereas loci that do not differ between sexes are considered autosomal. The method typically relies on statistical comparisons, such as chi‐square or Fisher's exact tests, and the presence/absence patterns of alleles across sexes. Then this function generates a plot displaying male and female heterozygosity at each locus or SNP and classifies loci into five categories: W‐linked or Y‐linked, sex‐biased, Z‐linked or X‐linked, gametologous and autosomal. Y or W‐specific alleles that are monomorphic on the X or Z chromosome manifest as loci that are consistently heterozygous in all individuals of the heterogametic sex and homozygous in all individuals of the homogametic sex. In an XX/XY system, gametologs—homologous, non‐recombining genes shared between the sex chromosomes—will appear as heterozygous in males and homozygous in females. Conversely, in a ZZ/ZW system, gametologous loci will be heterozygous in females and homozygous in males.

### 2.5 Sex Assignment

To assess the ability of sex‐linked SNP markers to predict the sex of individuals, we performed a cross‐validation procedure with 100 replicates on a dataset of 180 genotyped individuals at 72 SNPs (79 females, 101 males). At each replicate, 20% of individuals (*n* = 36) were randomly selected as a training set using stratified sampling by sex to preserve class proportions, with the remaining 80% (*n* = 144) serving as an independent test set. Genotypes (coded as allelic dosage 0/1/2) were first submitted to a Principal Component Analysis (PCA) computed on the training set, and individuals from the test set were then projected onto these axes. The first two principal components, explaining 95% of the genetic variance, were retained to avoid multicollinearity issues among markers. Sex classification was subsequently performed using two independent approaches: Linear Discriminant Analysis (LDA) and a k‐nearest neighbors' algorithm (k‐NN, k = 5). Performance was evaluated using overall accuracy, sensitivity (proportion of females correctly identified), and specificity (proportion of males correctly identified), averaged across all 100 replicates.

### 2.6 Characterizing the Sex Determination System

To define the sex determination system in *P. elephas* , we estimated the observed heterozygosity within individuals (Nei 1987) using *vcftools* with the function –het (Danecek et al. 2011) for each sex. This analysis was performed separately for the two marker types: SNPs associated with sex and identified by the *gl.sex.linked* function and putatively neutral markers. We used BLAST (Basic Local Alignment Search Tool) with an *e*‐value threshold of 10^−6^ to query the NCBI crustacean database to assess whether sequences in which sex‐linked markers were found corresponded to genomic regions previously associated with sex determination in other organisms.

### 2.7 Sex Assignment of Unsexed Individuals

We then tested the ability of these sex‐linked markers to infer the sex of 61 individuals whose sex was either undefined or not reported at the time of sampling. Using the sex information from the 180 sexed individuals, we assigned each identified cluster to a corresponding sex. The sex of the unsexed individuals was then inferred from their heterozygosity patterns. A major limitation of this approach is that the true sex of the 61 unsexed individuals remains unknown, meaning that our analysis primarily evaluates the power of the sex‐linked markers to assign individuals to a given sex cluster rather than directly validating their biological sex.

## 3 Results

### 3.1 Test for Sex‐Biased Dispersal Based on Putatively Neutral SNPs

A total of 8390 non‐outlier loci were identified and used for analyses in both females and males. The Loiselle relatedness index was identical for females (mean = −0.0002 ± 0.0070) and males (mean = −0.0002 ± 0.0071), with no significant difference between groups (ANOVA, *p*‐value = 0.48). Overall, relatedness values ranged from −0.028 to 0.05, indicating the absence of closely related individuals in the dataset (Figure S1).

**FIGURE 2:**
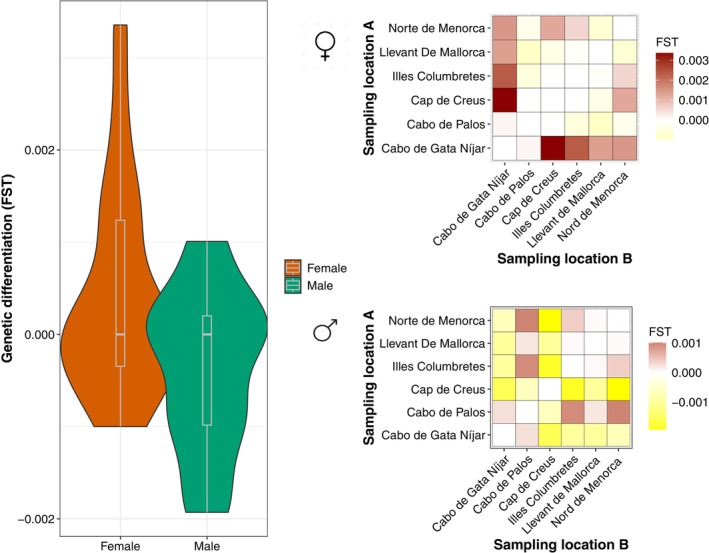
Genetic differentiation for female and male. Violin plot with boxplot of the *F*_*ST*_ index of genetic differentiation estimated according to Weir and Cockerham (1984) (left). The middle line represents the median and the box encompasses the interquartile range (IQR, 25th–75th percentiles). Heatmap showing the pairwise *F*_ST_ among all reserves for female (top) and male (bottom), separately (right).

Genetic differentiation between marine reserves was marginally higher in females than in males, with mean *F*_ST_ values of 0.0003 (range −0.001 to 0.003) and −0.0003 (range −0.0002 to 0.001), respectively. Yet, none of the *F*_ST_ values were significantly different (*p*‐value > 0.01). The overall mean *F*_ST_ across all markers (including both sexes) was 0.0002. In females, genetic variation followed a geographical structure along a north–south gradient, with Cabo de Gata Níjar in the south being the most genetically differentiated marine reserve. In contrast, genetic variation in males showed less geographic structuring (Figure 2). The AMOVA indicated that the components of genetic variance attributed to differences among sexes and among sampling locations were negative and not statistically significant (Table 2). Most of the genetic variation was therefore explained by within‐group differences among individuals rather than by structure associated with sex or geography. These results provide no evidence for genetic differentiation between males and females or among the sampled sites.

The *k*‐means clustering algorithm implemented in the Discriminant Analysis in Principal Component (DAPC) did not detect any genetic clusters. However, in females, the supervised DAPC, using sampling locations as priors, revealed that Cabo de Gata Níjar differed from the other marine reserves along the first axis, which accounted for 38.08% of the total genetic variation (Figure S2). No genetic clustering was observed in males using the supervised DAPC.

Our estimate of effective population size was twice as high for females (mean = 39,481, CI: 22,049–188,175) than for males (mean = 17,241, CI: 13,602–23,534, Table 3). However, the confidence intervals overlapped, suggesting that the difference in *N*_e_ between the sexes is not significant.

**TABLE 3:** Estimate of effective population size (Estimated *N*_e_) for *Palinurus elephas* females and males with lower and upper confidence intervals (CI low and CI high).

Sex	#samples	# of loci	Estimated Ne (MAF > 0.05)	CI low	CI high
Male	101	8930	17,241	13,602	23,534
Female	79	8930	39,481	22,049	188,175

### 3.2 Sex Determination System

**FIGURE 3:**
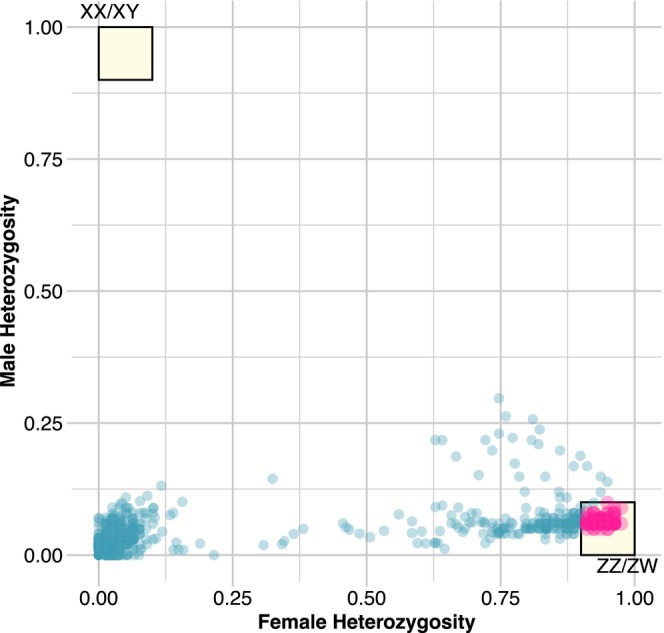
Identification of sex‐linked markers based on per‐locus heterozygosity patterns consistent with a ZZ/ZW sex determination system. Each point represents a SNP locus, plotted according to its observed heterozygosity in females (ZW, *x*‐axis) and males (ZZ, *y*‐axis). Sex‐linked markers are characterized by high heterozygosity in females (ZW) and low heterozygosity in males (ZZ), reflecting the hemizygous nature of the W‐linked region. Pale yellow boxes indicate the regions of the heterozygosity space in which sex‐linked markers were identified (*n* = 72).

**FIGURE 4:**
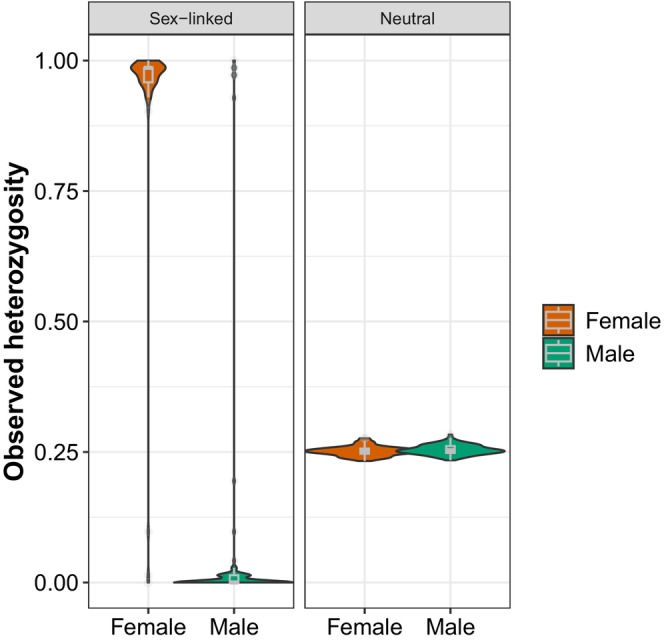
Observed heterozygosity compared between females and males according to the marker types used: Sex‐linked and putative neutral SNPs.

The 72 sex‐linked SNPs exhibited significant differences in heterozygosity between the sexes, with females displaying an excess of heterozygosity (*H*_o_ = 0.989 ± 0182) and males exhibiting a deficit of heterozygosity (*H*_o_ = 0.068 ± 0.231). This pattern is consistent with a ZZ/ZW sex determination system (Figure 3). It suggests that three females (pal_081, pal_074, pal_075) and four males (pal_spaS_018, pal_bal_134, pal_bal_128, pan_spaS_019) may have been misgendered, as their heterozygosity patterns did not match their recorded sex. We also examined heterozygosity patterns using the 8390 putatively neutral SNPs. As expected, no significant differences in heterozygosity between the sexes were observed for these markers (*p*‐value = 0.173, Figure 4) with comparable heterozygosity values between females (mean = 0.251, range: 0.013–0.679) and males (mean = 0.254, range: 0.019–0.653).

### 3.3 Sex Assignment

To evaluate the power of the sex‐linked markers to correctly assign biological sex, we performed a training/prediction approach on the 180 sexed individuals. We randomly withheld 20% of individuals (*n* = 36), defined a heterozygosity threshold on the remaining 80% (*n* = 144), and predicted the sex of the withheld individuals based on their observed heterozygosity across the 72 sex‐linked markers on 100 random replicates. This approach yielded an accuracy of 94.9%, supporting the reliability of our sex‐assignment method.

### 3.4 Sex‐Linked Markers

We used the 72 sex‐linked markers to infer the sex of the 61 individuals of unknown sex based on their heterozygosity. All the individuals were unambiguously assigned to one of the two groups, as each individual was either heterozygous at almost all 72 markers or homozygous at all 72 markers. As expected, the observed heterozygosity estimates differed significantly between the newly classified female and male groups (*p*‐value < 0.001), closely matching that of known females for the first group, with *H*_o_ of 0.952 (similarly to *H*_o_ = 0.989), while the second group had an average *H*_o_ of 0.042, similar to known males (*H*_o_ = 0.068). Based on these results, we identified 34 putative females and 27 putative males from the 61 unsexed individuals. Because our study system follows a ZW/ZZ sex‐determination mechanism, females are almost always heterozygous whereas males are homozygous.

The BLAST search yielded poor matches with the NCBI database. Of the 72 sex‐linked SNPs, only two SNPs were associated with a known gene (pancreas transcription factor), neither of which had functions directly related to sex determination.

## 4 Discussion

Theoretical and empirical studies suggest that male‐biased dispersal is generally favored in polygamous systems (reviewed in Li and Kokko 2019), yet research on sex‐biased dispersal in marine invertebrates remains scarce. Building on this framework, we leveraged a large panel of genomic markers to investigate sex‐biased dispersal in the vulnerable European spiny lobster, *Palinurus elephas* . Our study approaches this eco‐evolutionary puzzle by assessing key genetic analyses, from relatedness to effective population size, in both males and females.

### 4.1 Sex‐Biased Dispersal

The dominant explanation for sex‐biased dispersal stems from Greenwood's ideas (Greenwood 1980) about the success of philopatric versus dispersing individuals in securing mates or the resources to attract them. The philopatric sex gains a competitive advantage through familiarity with local resources and social structure, while the dispersing sex benefits from reduced kin competition and increased outbreeding. In species exhibiting strong homing and orientation abilities, philopatry may also increase survival because individuals are familiar with local shelters and habitat features. For species displaying marked day–night activity patterns, access to known shelters is critical for survival, and the presence of multiple shelters within a given area can increase reproductive success by facilitating repeated use of favorable breeding sites (e.g., Boles and Lohmann 2003). Sex‐biased dispersal has been demonstrated in 257 species of vertebrates and arthropods (Trochet et al. 2016). In our study, we did not detect a sex‐biased dispersal pattern, as there were no significant differences in relatedness and effective population size and genetic differentiation between the sexes. Although higher catchability of mature males was observed prior to reproduction, which could be explained by a higher mobility of males to find females to copulate (Goñi et al. 2003). Nevertheless, *F*‐statistics revealed different levels of population genetic structure between the sexes, with females exhibiting slightly greater and more geographically structured genetic differentiation. This population structure in females is consistent with the literature on genetic connectivity between the Atlantic and the Mediterranean, particularly the Almeria‐Oran front, where numerous species show pronounced shifts in allele frequencies (Patarnello et al. 2007). A similar pattern of population structure has been observed in the comber ( *Serranus cabrilla* ) in the same region as our study (Benestan et al. 2021), and in *P. elephas* across the Almeria‐Oran front near Cabo de Gata Níjar (Elphie et al. 2012). Furthermore, previous studies on sex‐biased dispersal in crustaceans suggest that females may exhibit philopatry (Durand et al. 2019; King et al. 2005), likely due to the parental care provided by brooding‐egg females, which is thought to promote female philopatry (Palaoro and Thiel 2020). Despite this, no clear relationship has been established between lobster movements, homing and orientation with respect to sex or size (Goñi et al. 2010). To refine our findings on the lack of sex‐biased dispersal in *P. elephas* , additional direct methods to quantify dispersal in the wild, such as precise geographic physical tagging studies, as an acoustic telemetry, would be valuable, as well as a larger sample size.

### 4.2 Sex‐Linked Markers for Monitoring Populations

Sex information is a critical factor to consider when delineating population structure, especially when establishing conservation and management units (Funk et al. 2012). The use of techniques such as restriction‐site associated DNA sequencing (RADseq) and related approaches has proven effective in identifying sex‐linked markers in a variety of organisms (Gamble and Zarkower 2014; Kafkas et al. 2015; Krueger‐Hadfield et al. 2020). As illustrated here, sex‐linked markers provide the opportunity to determine sex, which is particularly valuable when morphological identification is not possible, such as at juvenile stages.

The 72 sex‐linked markers identified in this study provide a robust basis for sex assignment based on individual heterozygosity patterns. In a cross‐validation framework applied to the 180 sexed individuals, this approach yielded an accuracy of 94.9%, supporting its reliability for applied use. While real time, boat‐side molecular sexing remains beyond current technological reach, laboratory‐based sex assignment using targeted SNP genotyping platforms such as Fluidigm EP1 or MassARRAY (Agena) is both feasible and cost‐effective, with per‐sample cost of approximately 10–20 € per individual and turnaround times of 24–48 h. The initial development of a dedicated SNP panel for *Palinurus elephas* would represent a onetime investment of approximately 20,000€, after which routine genotyping costs would decrease substantially through economies of scale. Such an approach would be particularly relevant for fisheries compliance monitoring, where conventional morphological sexing is often impractical, and for population management applications involving juveniles or archived specimens.

Determining the sex of *Palinurus elephas* juveniles is important in population and conservation studies. It allows researchers to assess sex ratios at recruitment, which is crucial for understanding population structure and predicting future reproductive potential. Sex identification also provides insight into potential sex‐biased dispersal patterns, helping to evaluate connectivity between populations and colonization of new habitats. Additionally, knowing the sex of juveniles facilitates genetic analyses, such as detecting sex‐linked loci and examining sex‐specific patterns of genetic diversity. Using sex‐linked markers, we were able to unambiguously assign sex to 100% of the unsexed individuals, highlighting their potential to improve resource management. From a management perspective, this information can guide conservation strategies and restocking programs by ensuring balanced sex ratios and protecting reproductive individuals effectively. This also reveals a crucial point: some individuals initially identified morphologically did not match the sex found using genetic markers, underlining the possibility of misidentification based on morphological traits. This mismatch highlights the limitations of visual sexing in this species and underscores the need for a reliable molecular tool for sex determination. In contrast, genetic markers offer a more reliable approach to sex determination. However, no functional annotation was found for these markers. The limited congruence with existing databases is likely due to the short length of the DArT sequences (69 base pairs), which can hinder accurate alignment and gene identification. This highlights the challenges of using short sequences for functional annotation and suggests that further efforts are needed to identify sex‐linked genes in *P. elephas* using alternative methods, such as longer sequence reads or targeted gene discovery approaches. More broadly, sex‐linked markers may be useful in species such as *P. elephas* , where fishery regulations may be sex‐specific (e.g., prohibition of harvesting females during the breeding season, or return males with high potential reproductive). These markers make it possible to identify the sex of individuals even from processed tissue samples, which is valuable for monitoring compliance with fisheries regulations and for populations management. Sexing individuals is also the first step in accurately assessing sex ratios, both in wild populations and in fisheries landings or aquaculture. This method holds great promise for improving management practices and ensuring the sustainable use of marine resources.

### 4.3 Sex Determination System

Sex determination systems (e.g., XX/XY, ZZ/ZW, hermaphroditism) can be diverse in some groups such as fish and invertebrates while being conserved in others, such as mammals (XX/XY) and birds (ZZ/ZW). In arthropods, and specifically in decapods, sex determination systems are particularly diverse (Chandler et al. 2018). In this study, we sampled the genome of *P. elephas* using DartSeq technology, identified sex‐linked markers likely located on sex chromosomes, and document for the first time a ZZ/ZW sex determination system in this species, with females being the heterogametic sex. ZZ/ZW systems have also been identified in several decapods such as crayfish ( *Cherax quadricarinatus* ), crab ( *Eriocheir sinensis* ) and shrimp (*Fenneropenaeus chinensis, Litopenaeus vannamei*, *Marsupenaeus japonicas*, and *Penaeus monodon* ; reviewed in Chandler et al. 2018), while the XX/XY sex determination system has been reported in the American lobster ( *Homarus americanus* ; Benestan et al. 2017; Moore and Benestan 2018). As a rule of thumb, crabs and lobsters mostly have an XX/XY system, while shrimps and crayfish have a ZZ/ZW system, with notable exceptions (Chandler et al. 2018). The fact that decapods are a highly diverse and old group with many independent radiations may facilitate multiple independent evolutions or transitions between sex determination systems (Ye et al. 2023). In contrast to these observations, we discovered a ZZ/ZW system in *Palinurus elephas* , a spiny lobster of the order Decapodea. Our results contribute to our understanding of the paradoxical diversity of sex determination systems in invertebrates, even within the same order (Decapodea).

## Author Contributions

**Laura Benestan:** conceptualization (lead), data curation (lead), formal analysis (lead), investigation (lead), methodology (lead), visualization (lead), writing – original draft (lead), writing – review and editing (lead). **Alicia Dalongeville:** investigation (supporting), methodology (supporting), writing – review and editing (equal). **Raquel Goñi:** resources (equal), validation (equal), writing – review and editing (equal). **Sandra Mallol:** resources (equal), writing – review and editing (equal). **David Díaz:** resources (equal), writing – review and editing (equal). **Oscar Puebla:** conceptualization (equal), project administration (equal), supervision (equal), writing – review and editing (equal). **Stéphanie Manel:** conceptualization (equal), project administration (equal), supervision (equal), writing – review and editing (equal).

## Funding

This research (RESERVEBENEFIT) was funded through the 2015–2016 BiodivERsA COFUND call for research proposals, with the National Funders ANR (France), Formas (Sweden), DLR (Germany), and AEI (Spain).

## Disclosure

All authors have read and approved the final version of the manuscript. The corresponding author agrees to be accountable for all aspects of the work in ensuring that questions related to the accuracy or integrity of any part of the work are appropriately investigated and resolved.

## Conflicts of Interest

The authors declare no conflicts of interest.

## Supporting information


**Figure S1:** Violin plot with boxplot of the estimated Loiselle relatedness index estimated for each sex separately (females in orange, males in green).
**Figure S2:** Discriminant Analysis of Principal Component (DAPC) conducted on 79 females using 8390 putatively neutral SNPs. Each point in the analysis represents an individual, with colors indicating the reserve to which each individual belongs. This approach helps visualize genetic differentiation and potential clustering based on geographic origin.
**Table S1:**. Filtering steps for the genetic data. The table indicates the number of loci remaining after each filtering step. The data were filtered with respect to minor allele frequency, sequencing coverage, missing data, linkage disequilibrium and selection (pcadapt).

## Data Availability

All code used for the analysis is available on https://github.com/laurabenestan/sex_lobster at Laura Benestan github page. We deposited all data on Dryad (https://doi.org/10.5061/dryad.sj3tx96gm). The metadata from our biological samples were also submitted to geOme under the Reservebenefit name (Deck et al. 2017).

## References

[ece373617-bib-0001] Allendorf, F. W. , P. A. Hohenlohe , and G. Luikart . 2010. “Genomics and the Future of Conservation Genetics.” Nature Reviews Genetics 11, no. 10: 697–709. 10.1038/nrg2844. 20847747

[ece373617-bib-0002] Babbucci, M. , S. Buccoli , A. Cau , et al. 2010. “Population Structure, Demographic History, and Selective Processes: Contrasting Evidences From Mitochondrial and Nuclear Markers in the European Spiny Lobster *Palinurus elephas* (Fabricius, 1787).” Molecular Phylogenetics and Evolution 56, no. 3: 1040–1050. 10.1016/j.ympev.2010.05.014. 20510378

[ece373617-bib-0003] Baguette, M. , T. G. Benton , and J. M. Bullock . 2012. “Multicausality of Dispersal: A Review.” In Dispersal Ecology and Evolution, edited by J. Clobert , M. Baguette , T. G. Benton , and J. M. Bullock . Oxford University Press. 10.1093/acprof:oso/9780199608898.001.0001.

[ece373617-bib-0004] Baines, C. B. , I. M. Ferzoco , and S. J. McCauley . 2017. “Sex‐Biased Dispersal Is Independent of Sex Ratio in a Semiaquatic Insect.” Behavioral Ecology and Sociobiology 71, no. 8: 119. 10.1007/s00265-017-2348-7.

[ece373617-bib-0005] Ball, L. , K. Shreves , M. Pilot , and A. E. Moura . 2017. “Temporal and Geographic Patterns of Kinship Structure in Common Dolphins ( *Delphinus delphis* ) Suggest Site Fidelity and Female‐Biased Long‐Distance Dispersal.” Behavioral Ecology and Sociobiology 71: 123. 10.1007/s00265-017-2351-z. 28794579 PMC5522516

[ece373617-bib-0006] Benestan, L. , K. Fietz , N. Loiseau , et al. 2021. “Restricted Dispersal in a Sea of Gene Flow.” Proceedings of the Royal Society B: Biological Sciences 288, no. 1951: 20210458. 10.1098/rspb.2021.0458. PMC8131118 34004134

[ece373617-bib-0007] Benestan, L. , J. S. Moore , B. J. G. Sutherland , et al. 2017. “Sex Matters in Massive Parallel Sequencing: Evidence for Biases in Genetic Parameter Estimation and Investigation of Sex Determination Systems.” Molecular Ecology 26, no. 24: 6767–6783. 10.1111/mec.14217. 28658525

[ece373617-bib-0008] Bevacqua, D. , P. Melià , M. C. Follesa , G. A. De Leo , M. Gatto , and A. Cau . 2010. “Body Growth and Mortality of the Spiny Lobster *Palinurus elephas* Within and Outside a Small Marine Protected Area.” Fisheries Research 106, no. 3: 543–549. 10.1016/j.fishres.2010.10.008.

[ece373617-bib-0073] Boles, L. C. , and K. J. Lohmann . 2003. “True Navigation and Magnetic Maps in Spiny Lobsters.” Nature 421: 60–63. 12511953 10.1038/nature01226

[ece373617-bib-0009] Cau, A. , A. Bellodi , R. Cannas , et al. 2019. “European Spiny Lobster Recovery From Overfishing Enhanced Through Active Restocking in Fully Protected Areas.” Scientific Reports 9, no. 1: 13025. 10.1038/s41598-019-49553-8. 31506533 PMC6737030

[ece373617-bib-0010] Cayuela, H. , Q. Rougemont , J. G. Prunier , et al. 2018. “Demographic and Genetic Approaches to Study Dispersal in Wild Animal Populations: A Methodological Review.” Molecular Ecology 27, no. 20: 3976–4010. 10.1111/mec.14848. 30152121

[ece373617-bib-0011] Chandler, J. C. , A. Elizur , and T. Ventura . 2018. “The Decapod Researcher's Guide to the Galaxy of Sex Determination.” Hydrobiologia 825, no. 1: 61–80. 10.1007/s10750-017-3452-4.

[ece373617-bib-0012] Chesser, R. K. , and R. J. Baker . 1996. “Effective Sizes and Dynamics of Uniparentally and Diparentally Inherited Genes.” Genetics 144, no. 3: 1225–1235. 10.1093/genetics/144.3.1225. 8913763 PMC1207614

[ece373617-bib-0013] Clobert, J. , M. Baguette , T. G. Benton , and J. M. Bullock . 2012. Dispersal Ecology and Evolution. Oxford University Press.

[ece373617-bib-0014] Clusa, M. , C. Carreras , L. Cardona , et al. 2018. “Philopatry in Loggerhead Turtles *Caretta caretta* : Beyond the Gender Paradigm.” Marine Ecology Progress Series 588: 201–213. 10.3354/meps12448.

[ece373617-bib-0015] Danecek, P. , A. Auton , G. Abecasis , et al. 2011. “The Variant Call Format and VCFtools.” Bioinformatics 27, no. 15: 2156–2158. 10.1093/bioinformatics/btr330. 21653522 PMC3137218

[ece373617-bib-0016] Day, J. , J. A. Clark , J. E. Williamson , C. Brown , and M. Gillings . 2019. “Population Genetic Analyses Reveal Female Reproductive Philopatry in the Oviparous Port Jackson Shark.” Marine and Freshwater Research 70, no. 7: 986–994. 10.1071/MF18255.

[ece373617-bib-0017] Deck, J. , M. R. Gaither , R. Ewing , et al. 2017. “The Genomic Observatories Metadatabase (GeOMe): A New Repository for Field and Sampling Event Metadata Associated With Genetic Samples.” PLoS Biology 15, no. 8: e2002925. 10.1371/journal.pbio.2002925. 28771471 PMC5542426

[ece373617-bib-0018] Do, C. , R. S. Waples , D. Peel , G. M. Macbeth , B. J. Tillett , and J. R. Ovenden . 2014. “NeEstimator v2: Re‐Implementation of Software for the Estimation of Contemporary Effective Population Size (Ne) From Genetic Data.” Molecular Ecology Resources 14, no. 1: 209–214. 10.1111/1755-0998.12157. 23992227

[ece373617-bib-0065] Dobson, F. S. 1982. “Competition for Mates and Predominant Juvenile Male Dispersal in Mammals.” Animal Behaviour 30, no. 4: 1183–1192. 10.1016/S0003-3472(82)80209-1.

[ece373617-bib-0019] Dudaniec, R. Y. , A. R. Carey , E. I. Svensson , B. Hansson , C. J. Yong , and L. T. Lancaster . 2022. “Latitudinal Clines in Sexual Selection, Sexual Size Dimorphism and Sex‐Specific Genetic Dispersal During a Poleward Range Expansion.” Journal of Animal Ecology 91, no. 6: 1104–1118. 10.1111/1365-2656.13488. 33759189

[ece373617-bib-0020] Durand, S. , F. Grandjean , I. Giraud , R. Cordaux , S. Beltran‐Bech , and N. Bech . 2019. “Fine‐Scale Population Structure Analysis in *Armadillidium vulgare* (Isopoda: Oniscidea) Reveals Strong Female Philopatry.” Acta Oecologica 101: 103478. 10.1016/j.actao.2019.103478.

[ece373617-bib-0021] Elphie, H. , G. Raquel , D. David , and P. Serge . 2012. “Detecting Immigrants in a Highly Genetically Homogeneous Spiny Lobster Population ( *Palinurus elephas* ) in the Northwest Mediterranean Sea.” Ecology and Evolution 2, no. 10: 2387–2396. 10.1002/ece3.349. 23145326 PMC3492767

[ece373617-bib-0022] Fietz, K. , E. Trofimenko , P. E. Guerin , et al. 2020. “New Genomic Resources for Three Exploited Mediterranean Fishes.” Genomics 112, no. 6: 4297–4303. 10.1016/j.ygeno.2020.06.041. 32629099

[ece373617-bib-0023] Follesa, M. C. , D. Cuccu , R. Cannas , A. Sabatini , A. M. Deiana , and A. Cau . 2009. “Movement Patterns of the Spiny Lobster *Palinurus elephas* (Fabricius, 1787) From a Central Western Mediterranean Protected Area.” Scientia Marina 73, no. 3: 499–506. 10.3989/scimar.2009.73n3499.

[ece373617-bib-0024] Funk, W. C. , J. K. McKay , P. A. Hohenlohe , and F. W. Allendorf . 2012. “Harnessing Genomics for Delineating Conservation Units.” Trends in Ecology & Evolution 27, no. 9: 489–496. 10.1016/j.tree.2012.05.012. 22727017 PMC4185076

[ece373617-bib-0025] Gamble, T. , and D. Zarkower . 2014. “Identification of Sex‐Specific Molecular Markers Using Restriction Site‐Associated DNA Sequencing.” Molecular Ecology Resources 14, no. 5: 902–913. 10.1111/1755-0998.12237. 24506574

[ece373617-bib-0026] Goñi, R. , R. Hilborn , D. Díaz , S. Mallol , and S. Adlerstein . 2010. “Net Contribution of Spillover From a Marine Reserve to Fishery Catches.” Marine Ecology Progress Series 400: 233–243. 10.3354/meps08419.

[ece373617-bib-0027] Goñi, R. , and D. Latrouite . 2005. “Review of the Biology, Ecology and Fisheries of *Palinurus* spp. Species of European Waters: *Palinurus elephas* (Fabricius, 1787) and *Palinurus mauritanicus* (Gruvel, 1911).” Cahiers de Biologie Marine 46, no. 2: 127–142.

[ece373617-bib-0028] Goñi, R. , A. Quetglas , and O. Reñones . 2003. “Differential Catchability of Male and Female European Spiny Lobster *Palinurus elephas* (Fabricius, 1787) in Traps and Trammelnets.” Fisheries Research 65, no. 1–3: 295–307. 10.1016/j.fishres.2003.09.021.

[ece373617-bib-0029] Goudet, J. , and T. Jombart . 2015. Estimation and Tests of Hierarchical F‐Statistics. R Core Team.

[ece373617-bib-0030] Goudet, J. , N. Perrin , and P. Waser . 2002. “Tests for Sex‐Biased Dispersal Using Bi‐Parentally Inherited Genetic Markers.” Molecular Ecology 11, no. 6: 1103–1114. 10.1046/j.1365-294X.2002.01496.x. 12030985

[ece373617-bib-0031] Greenwood, P. J. 1980. “Mating Systems, Philopatry and Dispersal in Birds and Mammals.” Animal Behaviour 28, no. 4: 1140–1162. 10.1016/S0003-3472(80)80103-5.

[ece373617-bib-0032] Gruber, B. , P. J. Unmack , O. F. Berry , and A. Georges . 2018. “Dartr: An r Package to Facilitate Analysis of SNP Data Generated From Reduced Representation Genome Sequencing.” Molecular Ecology Resources 18, no. 3: 691–699. 10.1111/1755-0998.12745. 29266847

[ece373617-bib-0033] Hutchings, J. A. , and L. Gerber . 2002. “Sex‐Biased Dispersal in a Salmonid Fish.” Proceedings of the Royal Society of London, Series B: Biological Sciences 269: 2487–2493. 10.1098/rspb.2002.2176. PMC1691174 12495493

[ece373617-bib-0034] Johnstone, R. A. , M. A. Cant , and J. Field . 2012. “Sex‐Biased Dispersal, Haplodiploidy and the Evolution of Helping in Social Insects.” Proceedings of the Royal Society B: Biological Sciences 279, no. 1729: 787–793. 10.1098/rspb.2011.1257. PMC3248733 21795270

[ece373617-bib-0035] Jombart, T. , S. Devillard , and F. Balloux . 2010. “Discriminant Analysis of Principal Components: A New Method for the Analysis of Genetically Structured Populations.” BMC Genetics 11: 94. 10.1186/1471-2156-11-94. 20950446 PMC2973851

[ece373617-bib-0036] Kafkas, S. , M. Khodaeiaminjan , M. Güney , and E. Kafkas . 2015. “Identification of Sex‐Linked SNP Markers Using RAD Sequencing Suggests ZW/ZZ Sex Determination in *Pistacia vera* L.” BMC Genomics 16, no. 1: 98. 10.1186/s12864-015-1326-6. 25765114 PMC4336685

[ece373617-bib-0037] Kilian, A. , P. Wenzl , E. Huttner , et al. 2012. “Diversity Arrays Technology: A Generic Genome Profiling Technology on Open Platforms.” Methods in Molecular Biology 888: 67–89. 10.1007/978-1-61779-870-2_5. 22665276

[ece373617-bib-0038] King, T. L. , M. S. Eackles , A. P. Spidle , and H. J. Brockmann . 2005. “Regional Differentiation and Sex‐Biased Dispersal Among Populations of the Horseshoe Crab *Limulus polyphemus* .” Transactions of the American Fisheries Society 134, no. 2: 441–465. 10.1577/t04-023.1.

[ece373617-bib-0039] Krueger‐Hadfield, S. A. , B. A. Flanagan , O. Godfroy , et al. 2020. “Using RAD‐Seq to Develop Sex‐Linked Markers in a Haplodiplontic Alga.” Journal of Phycology 57: 279–294. 10.1111/jpy.13088. 33098662

[ece373617-bib-0040] Li, X. Y. , and H. Kokko . 2019. “Sex‐Biased Dispersal: A Review of the Theory.” Biological Reviews 94, no. 2: 721–736. 10.1111/brv.12475. 30353655 PMC7379701

[ece373617-bib-0042] Luu, K. , E. Bazin , and M. G. B. Blum . 2017. “Pcadapt: An R Package to Perform Genome Scans for Selection Based on Principal Component Analysis.” Molecular Ecology Resources 17, no. 1: 67–77. 10.1111/1755-0998.12592. 27601374

[ece373617-bib-0043] Meirmans, P. G. , and P. H. Van Tienderen . 2004. “Genotype and Genodive: Two Programs for the Analysis of Genetic Diversity of Asexual Organisms.” Molecular Ecology Notes 4, no. 4: 792–794. 10.1111/j.1471-8286.2004.00770.x.

[ece373617-bib-0044] Moland, E. , R. Goñi , B. J. Hanns , and N. T. Shears . 2025. “Recovery of Crustacean Populations in Protected Areas.” In Ecology and Conservation, edited by L. Gutow and M. Thiel , vol. 10. Oxford University Press. 10.1093/oso/9780197768242.001.0001.

[ece373617-bib-0045] Möller, L. M. , and L. B. Beheregaray . 2004. “Genetic Evidence for Sex‐Biased Dispersal in Resident Bottlenose Dolphins ( *Tursiops aduncus* ).” Molecular Ecology 13: 1607–1612. 10.1111/j.1365-294X.2004.02137.x. 15140103

[ece373617-bib-0046] Moore, J. S. , and L. Benestan . 2018. “Let's Talk About Sex: A Rigorous Statistical Framework to Assign the Sex of Individuals From Reduced‐Representation Sequencing Data.” Molecular Ecology Resources 18, no. 2: 191–193. 10.1111/1755-0998.12761. 29575750

[ece373617-bib-0047] Muñoz, A. , R. Goñi , C. Linares , et al. 2021. “Exploration of the Inter‐Annual Variability and Multi‐Scale Environmental Drivers of European Spiny Lobster, *Palinurus elephas* (Decapoda: Palinuridae) Settlement in the NW Mediterranean.” Marine Ecology 42, no. 3: e12654.

[ece373617-bib-0048] Nei, M. 1987. Molecular Evolutionary Genetics. Columbia University Press. 10.7312/nei-92038.

[ece373617-bib-0049] Nykänen, M. , E. Dillane , A. Englund , et al. 2018. “Quantifying Dispersal Between Marine Protected Areas by a Highly Mobile Species, the Bottlenose Dolphin, *Tursiops truncatus* .” Ecology and Evolution 8, no. 18: 9241–9258. 10.1002/ece3.4343. 30377497 PMC6194238

[ece373617-bib-0050] Palaoro, A. V. , and J. Beermann . 2020. “Overview of the Mating Systems of Crustacea.” In Reproductive Biology: The Natural History of the Crustacea, vol. 6, 275–304. Oxford University Press. 10.1093/oso/9780190688554.003.0010.

[ece373617-bib-0051] Palaoro, A. v. , and M. Thiel . 2020. “‘The Caring Crustacean’: An Overview of Crustacean Parental Care.” In Reproductive Biology: The Natural History of the Crustacea, vol. 6. Oxford University Press. 10.1093/oso/9780190688554.003.0005.

[ece373617-bib-0052] Palero, F. , P. Abelló , E. Macpherson , M. Beaumont , and M. Pascual . 2011. “Effect of Oceanographic Barriers and Overfishing on the Population Genetic Structure of the European Spiny Lobster ( *Palinurus elephas* ).” Biological Journal of the Linnean Society 104, no. 2: 407–418. 10.1111/j.1095-8312.2011.01728.x.

[ece373617-bib-0053] Pardini, A. T. , C. S. Jones , L. R. Noble , et al. 2001. “Sex‐Biased Dispersal of Great White Sharks.” Nature 412: 139–140. 10.1038/35084125. 11449258

[ece373617-bib-0054] Patarnello, T. , F. A. M. J. Volckaert , and R. Castilho . 2007. “Pillars of Hercules: Is the Atlantic‐Mediterranean Transition a Phylogeographical Break?” Molecular Ecology 16, no. 21: 4426–4444. 10.1111/j.1365-294X.2007.03477.x. 17908222

[ece373617-bib-0055] Peres, P. , A. Ferreira , G. Machado , M. Azevedo‐Silva , S. Siqueira , and F. Leite . 2021. “Sex‐Biased Dispersal Depends on the Spatial Scale in a Tube‐Building Amphipod.” Marine Ecology Progress Series 658: 135–148. 10.3354/meps13552.

[ece373617-bib-0056] Phillips, N. M. , F. Devloo‐Delva , C. McCall , and T. S. Daly‐Engel . 2021. “Reviewing the Genetic Evidence for Sex‐Biased Dispersal in Elasmobranchs.” Reviews in Fish Biology and Fisheries 31, no. 4: 821–841. 10.1007/s11160-021-09673-9.

[ece373617-bib-0057] Pike, V. L. , C. K. Cornwallis , and A. S. Griffin . 2021. “Why Don't All Animals Avoid Inbreeding?” Proceedings of the Royal Society B: Biological Sciences 288, no. 1956: 20211045. 10.1098/rspb.2021.1045. PMC8334842 34344184

[ece373617-bib-0058] Portnoy, D. S. , J. B. Puritz , C. M. Hollenbeck , J. Gelsleichter , D. Chapman , and J. R. Gold . 2015. “Selection and Sex‐Biased Dispersal in a Coastal Shark: The Influence of Philopatry on Adaptive Variation.” Molecular Ecology 24, no. 23: 5877–5885. 10.1111/mec.13441. 26518727

[ece373617-bib-0059] Prugnolle, F. , and T. de Meeus . 2002. “Inferring Sex‐Biased Dispersal From Population Genetic Tools: A Review.” Heredity 88: 161–165. 10.1038/sj.hdy.6800060. 11920116

[ece373617-bib-0060] Pusey, A. E. 1987. “Sex‐Biased Dispersal and Inbreeding Avoidance in Birds and Mammals.” Trends in Ecology & Evolution 2, no. 10: 295–299. 10.1016/0169-5347(87)90081-4. 21227869

[ece373617-bib-0061] Quetglas, A. , A. Gaamour , O. Reñones , et al. 2004. “Common Spiny Lobster ( *Palinurus elephas* Fabricius 1787) Fisheries in the Western Mediterranean: A Comparison of Spanish and Tunisian Fisheries.” Bolleti de La Societat D'historia Natural de Les Balears 47: 63–80.

[ece373617-bib-0062] Robledo‐Ruiz, D. A. , L. Austin , J. N. Amos , et al. 2023. “Easy‐To‐Use R Functions to Separate Reduced‐Representation Genomic Datasets Into Sex‐Linked and Autosomal Loci, and Conduct Sex Assignment.” Molecular Ecology Resources 25: e13844. 10.1111/1755-0998.13844. 37526650 PMC12142712

[ece373617-bib-0063] Saastamoinen, M. , G. Bocedi , J. Cote , et al. 2018. “Genetics of Dispersal.” Biological Reviews 93, no. 1: 574–599. 10.1111/brv.12356. 28776950 PMC5811798

[ece373617-bib-0064] Shaw, R. E. , S. C. Banks , and R. Peakall . 2018. “The Impact of Mating Systems and Dispersal on Fine‐Scale Genetic Structure at Maternally, Paternally and Biparentally Inherited Markers.” Molecular Ecology 27, no. 1: 66–82. 10.1111/mec.14433. 29154412

[ece373617-bib-0066] Teske, P. R. , I. Papadopoulos , N. P. Barker , and C. D. McQuaid . 2012. “Mitochondrial DNA Paradox: Sex‐Specific Genetic Structure in a Marine Mussel ‐ Despite Maternal Inheritance and Passive Dispersal.” BMC Genetics 13: 45. 10.1186/1471-2156-13-45. 22694765 PMC3465189

[ece373617-bib-0067] Trochet, A. , E. A. Courtois , V. M. Stevens , and M. Baguette . 2016. “Evolution of Sex‐Biased Dispersal.” Quarterly Review of Biology 91, no. 3: 297. 29558614 10.1086/688097

[ece373617-bib-0068] Wang, J. 2017. “Estimating Pairwise Relatedness in a Small Sample of Individuals.” Heredity 119, no. 5: 302–313. 10.1038/hdy.2017.52. 28853716 PMC5637371

[ece373617-bib-0069] Weir, B. S. , and C. C. Cockerham . 1984. “Estimating F‐Statistics for the Analysis of Population Structure.” Evolution 38, no. 6: 1358–1370. 10.1111/j.1558-5646.1984.tb05657.x. 28563791

[ece373617-bib-0070] Whitlock, M. C. , and N. H. Barton . 1997. “The Effective Size of a Subdivided Population.” Genetics 146, no. 1: 427–441. 10.1093/genetics/146.1.427. 9136031 PMC1207958

[ece373617-bib-0071] Wright, S. 1931. “Evolution in Mendelian Populations.” Bulletin of Mathematical Biology 52, no. 1–2: 241–295. 10.1007/BF02459575. 2185860

[ece373617-bib-0072] Ye, Z. , T. Bishop , Y. Wang , R. Shahriari , and M. Lynch . 2023. “Evolution of Sex Determination in Crustaceans.” Marine Life Science & Technology 5, no. 1: 1–11. 10.1007/s42995-023-00163-4. 37073332 PMC10077267

