# Supplementary material for: Investigating Sex‐Biased Dispersal in a Vulnerable Marine Invertebrate, the European Spiny Lobster (Palinurus elephas)
Source: Ecol Evol. 2026 Jun 7;16(6):e73617. doi: 10.1002/ece3.73617 (PMC13242979; doi:10.1002/ece3.73617)
Supplement: Supplementary file 1 — Figure S1: Violin plot with boxplot of the estimated Loiselle relatedness index estimated for each sex separately (females in orange, males in green). Figure S2: Discriminant Analysis of Principal Component (DAPC) conducted on 79 females using 8390 putatively neutral SNPs. Each point in the analysis represents an individual, with colors indicating the reserve to which each individual belongs. This approach helps visualize genetic differentiation and potential clustering based on geographic origin. Table S1:. Filtering steps for the genetic data. The table indicates the number of loci remaining after each filtering step. The data were filtered with respect to minor allele frequency, sequencing coverage, missing data, linkage disequilibrium and selection (pcadapt). [file ECE3-16-e73617-s001.docx]

**Supplementary figures**

**Figure S1.** Violin plot with boxplot of the estimated Loiselle relatedness index estimated for each sex separately (females in orange, males in green).

**Figure S2.** Discriminant Analysis of Principal Component (DAPC) conducted on 79 females using 8,390 putatively neutral SNPs. Each point in the analysis represents an individual, with colors indicating the reserve to which each individual belongs. This approach helps visualize genetic differentiation and potential clustering based on geographic origin.

**Table S1**. Filtering steps for the genetic data. The table indicates the number of loci remaining after each filtering step. The data were filtered with respect to minor allele frequency, sequencing coverage, missing data, linkage disequilibrium and selection (pcadapt).

| **Filtering step** | ***Palinurus elephas*** |
| --- | --- |
| *All* | 126,598 |
| MAF 0.05 | 83,364 |
| Coverage 10x-100x | 78,489 |
| Missingness 0.2 | 57,113 |
| Linkage disequilibrium | 56,938 |
| R^2^ < 0.8 |  |
| Neutral dataset  from Benestan et al., 2021 | 25,230 |
| Subset of 180 individuals | 25,225 |
| 95% genotyping rate | 23,814 |
| MAF 0.05 | 9,931 |
| Hardy-Weinberg equilibrium | 8,390 |
| Heterozygosity < 06 | 8,390 |
